# Tenacibaculosis caused by *Tenacibaculum maritimum*: Updated knowledge of this marine bacterial fish pathogen

**DOI:** 10.3389/fcimb.2022.1068000

**Published:** 2023-01-06

**Authors:** Mahmoud Mabrok, Abdelazeem M. Algammal, Elayaraja Sivaramasamy, Helal F. Hetta, Banan Atwah, Saad Alghamdi, Aml Fawzy, Ruben Avendaño-Herrera, Channarong Rodkhum

**Affiliations:** ^1^ Department of Fish Diseases and Management, Faculty of Veterinary Medicine, Suez Canal University, Ismailia, Egypt; ^2^ Department of Veterinary Microbiology, Faculty of Veterinary Science, Chulalongkorn University, Bangkok, Thailand; ^3^ Center of Excellence in Fish Infectious Diseases (CE FID), Faculty of Veterinary Science, Chulalongkorn University, Bangkok, Thailand; ^4^ Department of Bacteriology, Mycology and Immunology, Faculty of Veterinary Medicine, Suez Canal University, Ismailia, Egypt; ^5^ Department of Medical Microbiology and Immunology, Faculty of Medicine, Assuit University, Assuit, Egypt; ^6^ Laboratory Medicine Department, Faculty of Applied Medical Sciences, Umm Al-Qura University, Makkah, Saudi Arabia; ^7^ Directorate of Veterinary Medicine, Ismailia, Egypt; ^8^ Laboratorio de Patología de Organismos Acuáticos y Biotecnología Acuícola, Facultad de Ciencias Biológicas, Universidad Andrés Bello, Viña del Mar, Chile; ^9^ Interdisciplinary Center for Aquaculture Research (INCAR), Viña del Mar, Chile; ^10^ Centro de Investigación Marina Quintay (CIMARQ), Universidad Andrés Bello, Quintay, Chile

**Keywords:** *Tenacibaculum maritimum*, tenacibaculosis, pathogenicity, marine fish, aquaculture

## Abstract

Tenacibaculosis occurs due to the marine bacterial pathogen *Tenacibaculum maritimum*. This ulcerative disease causes high mortalities for various marine fish species worldwide. Several external clinical signs can arise, including mouth erosion, epidermal ulcers, fin necrosis, and tail rot. Research in the last 15 years has advanced knowledge on the traits and pathogenesis mechanisms of *T. maritimum*. Consequently, significant progress has been made in defining the complex host-pathogen relationship. Nevertheless, tenacibaculosis pathogenesis is not yet fully understood. Continued research is urgently needed, as demonstrated by recent reports on the re-emerging nature of tenacibaculosis in salmon farms globally. Current sanitary conditions compromise the development of effective alternatives to antibiotics, in addition to hindering potential preventive measures against tenacibaculosis. The present review compiles knowledge of *T. maritimum* reported after the 2006 review by Avendaño-Herrera and colleagues. Essential aspects are emphasized, including antigenic and genomic characterizations and molecular diagnostic procedures. Further summarized are the epidemiological foundations of the *T. maritimum* population structure and elucidations as to the virulence mechanisms of pathogenic isolates, as found using biological, microbiological, and genomic techniques. This comprehensive source of reference will undoubtable serve in tenacibaculosis prevention and control within the marine fish farming industry. Lastly, knowledge gaps and valuable research areas are indicated as potential guidance for future studies.

## 1 Introduction

Aquaculture is a prominent and promising food-production industry, globally providing animal-protein sources and helping to cover food shortage gaps arising from population overshoots (Subasinghe et al., 2009). Approximately 17% of animal protein and 7% of all protein for human consumption is derived from fish; indeed, more than 3.3 million people rely on fish for up to 20% of average per capita animal-protein intake (FAO, 2020). Increasing demand requires increased fish production, a particularly acute situation considering the recession of fisheries in recent decades (Molenaar and Caddell, 2019). New technologies are helping to propel the industry, including shrimp and, specifically, fish production (Kumar and Engle, 2016). However, more intensive farming may negatively impact culturing waters, as associated with environmental pollution and the emergence and spread of aquatic pathogens (Leung and Bates, 2013). Water-borne diseases are, a critical issue facing the global aquaculture industry (Melba et al., 2021). Aquatic diseases are not a singular event but, rather, one of a linked chain of events involving interactions among pathogenic microorganisms, aquatic species, and environmental conditions (Lieke et al., 2020).

Relatively few pathogenic bacteria account for most of the financial losses incurred by the industry. However, new “emerging” diseases arise biannually and constantly impose health challenges limiting fish production. Classically, pathogenic bacteria are classified as extracellular, facultative intracellular, and obligate intracellular (Silva, 2012). The survival and dispersion of facultative bacteria are uncertain, particularly as environmental conditions worsen. By contrast, most pathogenic microbes regularly exist as assembled colonies on the host or freely live in the aquatic environment (Hassan et al., 2021). Interestingly, all bacterial types could turn pathogenic if fish immunity is compromised (Kirjusina et al., 2007).

Prominent among the causes for economic losses in global aquaculture are emerging diseases caused by *Tenacibaculum* species (Family *Flavobacteriaceae*, Phylum Bacteroidetes). Most available studies involving *Tenacibaculum* species associate disease in marine fish with infection caused by *Tenacibaculum maritimum* (see review by Avendaño-Herrera et al., 2006a). This disease was originally described as a *Flexibacter* infection in association with mortalities among farmed red (*Pagrus major*) and blackhead (*Acanthopagrus schlegelii*) seabream in Japan (Masumura and Wakabayashi, 1977). From 1977 to 2006, the disease was variably termed in relation to external lesions, fish species, and fish age. These changes finally included a taxonomic name change for the bacterium (see reviews by Bernardet, 1998; Avendaño-Herrera et al., 2006a).

*Tenacibaculum* spp. infections are today commonly referred to as marine tenacibaculosis or, simply, tenacibaculosis, a term originally coined to describe the ulcerative disease caused by the filamentous *T. maritimum* bacterium (Avendaño-Herrera et al., 2006a). Tenacibaculosis is generally associated with gross lesions on the body, including ulcerative and/or necrotic skin lesions, an eroded/hemorrhagic mouth, frayed fins, and tail rot (Avendaño-Herrera et al., 2006a; Elanco, 2018). Despite the association between *T. maritimum* isolation and the aforementioned clinical signs, consensus as to the primary or opportunistic nature of this pathogen is lacking. Research by numerous groups in recent decades has significantly advanced understandings of the characteristics and pathogenesis of *T. maritimum*, thereby helping to define complex host-pathogen relationships (Elanco, 2018). However, tenacibaculosis pathogenesis is still not fully elucidated.

Most published reviews on *T. maritimum* report evidence existing prior to 2006. Therefore, the purpose of the present review is to compile and summarize knowledge obtained on *T. maritimum* within the last 15 years. Focus is given to phenotypic, antigenic, and genomic characterizations and to recent molecular diagnostic procedures. Additionally summarized are the epidemiological foundations of the *T. maritimum* population structure (i.e., host and geographical distribution). Finally reported are findings elucidating the virulence mechanisms of pathogenic *T. maritimum* isolates through biological, microbiological, and genomic techniques. The compilation of this information will prove invaluable for tenacibaculosis prevention and control in marine fish farming. Lastly, emphasis given to still existing knowledge gaps and valuable research areas will serve as guidance for future investigative efforts.

## 2 Description of *Tenacibaculum maritimum*

The original taxonomic classification of the previously mentioned *Flexibacter* bacterium was a topic of controversy and confusion until Suzuki et al. (2001). This research reclassified said bacterium as *T. maritimum*, in addition to adding *Tenacibaculum ovolyticum* and two other species to the newly formed genus. Today, *T. maritimum* is the type species of the *Tenacibaculum* genus, which includes 33 validly and 3 invalidly published species (https://www.bacterio.net/genus/tenacibaculum). All are exclusively found in marine environments attached to or associated with the surface of marine organisms such as macroalgae, various invertebrates, and fish. In addition to *T. maritimum*, seven other *Tenacibaculum* fish pathogens have been identified – *T. ovolyticum* from Atlantic halibut (*Hippoglossus hippoglossus*) (Hansen et al., 1992; Avendaño-Herrera et al., 2022a); *T. discolor* from European and Asian sea bass (*Dicentrarchus labrax* and *Lates calcarifer*) (Piñeiro-Vidal et al., 2008a); *T. soleae* from Senegalese sole (*Solea senegalensis*) (Piñeiro-Vidal et al., 2008b); *T. dicentrarchi* from Atlantic salmon (*Salmo salar*) (Piñeiro-Vidal et al., 2012; Avendaño-Herrera et al., 2016); *T. finnmarkense* from rainbow trout (*Oncorhynchus mykiss*) (Olsen et al., 2020); and *T. singaporense* (Miyake et al., 2020) and *T. piscium* (Olsen et al., 2020; Avendaño-Herrera et al., 2022b) from Corkwing wrasse (*Symphodus melops*). Hereinafter, this review will focus only on *T. maritimum*.

The physical traits of *T. maritimum* are as follows: filamentous; Gram-negative; usually sized 2 – 30 µm in length with a diameter of 0.5 µm; gliding motility; and positive growth on media containing ≥ 10% seawater (i.e., no growth with only NaCl as the only added salt) (Suzuki et al., 2001). Several traits are common to the *Tenacibaculum* genus, including the production of a primarily zeaxanthin yellow carotenoid pigment, flexirubin-type pigment are absent; the major respiratory quinone is menaquinone-6 and are catalase- and -oxidase positive (Suzuki, 2015). As a point of difference compared to other *Tenacibaculum* species, including *T. ovolyticum* (Hansen et al., 1992) and *T. finnmarkense* (Olsen et al., 2020), *T. maritimum* absorb Congo red on solid agar when some colonies on agar are directly flooding with drops of a 0 ± 01% aqueous solution of the dye (Avendaño-Herrera et al., 2004a).

Intraspecific phenotypic similarities among *T. maritimum* isolates may aid in taxonomic identification when compared to other *Tenacibaculum* species pathogenic to fish. Indeed, some of the above-mentioned morphotype and physiological properties can serve as initial tube and plate analyses for species differentiation, in addition to the application of traditional, ready-made identification systems (e.g., API ZYM and API 50 CH) (Kolygas et al., 2012; Yardimci and Timur, 2016; Fernández-Álvarez and Santos, 2018). However, API results are not always identical due to factors such as the inoculum, isolate, and incubation temperature, among others. For example, differences are reported for tests on nitrate reduction and hydrogen-sulfide production (Avendaño-Herrera et al., 2004a). The phenotypic and biochemical characteristics used for the precise detection and differentiation of *T. maritimum* against other fish pathogens are given in **Table 1**. The complete genome sequence of the type strain NCIMB 2154^T^ consists of a circular chromosome of 3,435,971 base pairs with 2,866 predicted protein-coding genes, excluding any plasmid (Pérez-Pascual et al., 2017). Some of these genes are implicated in *T. maritimum* virulence and pathogenicity, as discussed in section 8 *Virulence Factors*.

**Table 1:** Differential phenotypic and biochemical characteristics of the type strains for *Tenacibaculum* species associated with fish diseases.

Characteristic	*T. maritimum*	*T. ovolyticum*	*T. singaporense*	*T. discolor*	*T. piscium*	*T. soleae*	*T. dicentrarchi*	*T. finnmarkense*
**Colony morphology**	**Uneven edge, pale yellow**	**Regular edge, pale yellow**	**Orange witd circular shape**	**Uneven edge, bright yellow**	**Circular, bright yellow**	**Circular, yellow**	**Uneven edge, pale yellow**	**Undulating margin, yellow**
**Temperature (°C)**	15-34	4-25	20-45	14-38	4-25	14-30	4-30	2-20
**Congo Red Absorption**	+	nt	nt	nt	W	nt	+	–
**pH range**	5.9-8.6	6-10	5-9	6-8	4-10	5-10	6-8	4-9
**Flexirubin type pigment**	–	–	–	–	nt	–	–	–
**Tween 80 degradation**	+	+	nt	–	–	–	V	–
**Starch hydrolysis**	+	+	+	–	–	–	–	–
**Proline utilization**	–	–	nt	+	W	–	–	+
**Glutamate utilization**	W	–	nt	+	–	–	–	+
**Growth with seawater (%)**	30-100	70-100	30-100	30-100	10-100	55-100	30-100	50-100
**Trypsin**	+	nt	–	+	–	–	–	nt
**A-Chymotrypsin**	+	nt	W	+	–	–	–	–
**Nitrate reduction**	+	+	+	+	–	+	nt	+

Data are from Hansen et al. (1992); Piñeiro-Vidal et al. (2008a); Piñeiro-Vidal et al. (2008b); Piñeiro-Vidal et al. (2012); Miyake et al. (2020), and Olsen et al. (2020). +, positive; –, negative; V, variable; nt, not tested; and W, weak.

## 3 Antigenic and genetic characterization of *Tenacibaculum maritimum*

Various typing methods have been applied in epidemiological studies of *T. maritimum*, including the slide agglutination test; the dot-blot and immunoblotting assays; multiplex PCR (mPCR) serotyping; the randomly amplified polymorphic DNA (RAPD) method; the multi-locus sequence analysis (MLSA); and assembled draft genomes, among others. These methods confirm the existence of intra-specific diversity among *T. maritimum* isolates. In the early 2000s, three serotypes were identified through immunoblot analyses of lipopolysaccharides with varying degrees of relation to host fish species (Avendaño-Herrera et al., 2004a; Avendaño-Herrera et al., 2005a). Subsequent serotyping research describes up to four serotypes in *T. maritimum* (Piñeiro-Vidal et al., 2007; Fernández-Álvarez and Santos, 2018). This antigenic diversity makes it difficult to select candidate strain(s) for intra-species vaccine development, as described in section *9 Treatment and Vaccination Programs for *T. maritimum**.

Despite proposed serotyping schemes for *T. maritimum*, several disadvantages exist for conventional mammal-antisera-based serotyping, including sera availability and result interpretations (Gorski, 2021). These difficulties have spurred the development of new molecular techniques, such as those proposed by Whitfield et al. (2020), where determination of the O-antigen structure allows for differentiating serotypes. This method is based on genes coding for the biosynthesis of the O-antigen, which are often located in a single genomic cluster (i.e., the O-AGC) and fall into one of the following three classes: (i) nucleotide sugar biosynthesis genes; (ii) sugar transferase genes; or (iii) genes required for O-unit translocations, chain synthesis, and chain-length determination. Application of this knowledge is evidenced by Lopez et al. (2022a), a study that identified the O-AGC gene cluster in *T. maritimum* using a set of recently published *T. maritimum* genomes (Pérez-Pascual et al., 2017; Bridel et al., 2020) and primers specifically designed for the O-antigens of representative isolates of the defined serotypes (Avendaño-Herrera et al., 2004a; Avendaño-Herrera et al., 2005a). These breakthroughs allowed Lopez et al. (2022a) to develop an mPCR-based serotyping scheme that combines traditional serotyping with a genome-wide association study.

At the genetic level, cluster analyses of the RAPD-PCR profiles for 29 isolates and 3 reference strains of *T. maritimum* showed that, regardless of the oligonucleotide primer employed, the isolates and strains were separated into two main groups that strongly correlated with the host species and/or the described O serotypes (Avendaño-Herrera et al., 2004a; Avendaño-Herrera et al., 2004b). Elucidation of the *T. maritimum* genome through next generation sequencing techniques has allowed for (i) the design of primers directed at constitutive genes of the pathogen and (ii) the characterization of population diversity. While an advancement, these evolved developments naturally limited the use of random amplification techniques, such as those previously proposed by Avendaño-Herrera et al. (2004a), because they are based on a comparison of amplification patterns. These previous limitations have been overcome by Habib et al. (2014) through the application of MLSA to 114 isolates representatives of the *Tenacibaculum* genus, including *T. maritimum* isolates reflecting the global diversity of this species. The proposed method is based on eleven loci located within single-copy protein-coding genes conserved across the *Flavobacteriaceae* family were used to design generic degenerated PCR primers for the *Tenacibaculum* genus. Habib et al. (2014) highlighted the existence of a cohesive *T. maritimum* group and recommended the use thereof to monitor infections caused by *Tenacibaculum* species. However, not all primer sets given by Habib et al. (2014) had the same efficiency during amplification, leading Småge et al. (2016a) to propose the use of other primer sequences targeting five genes.

Confirmation of the MLSA scheme as a powerful tool for taxonomic affiliation and isolate identification of *T. maritimum* has been demonstrated with field isolates (Småge et al., 2016a; Valdes et al., 2021; Lopez et al., 2022a). Such power has even been shown for the pathogen *T. dicentrarchi* (Avendaño-Herrera et al., 2016; Klakegg et al., 2019) and several pathogenic species that have yet to be described (Olsen et al., 2017). In this sense, Frisch et al. (2018a) noted that genotyping in the Canadian region increased their understandings of the genetic profile of *T. maritimum*. These authors reported two new sequence types among Western Canadian isolates of *T. maritimum*. Despite the known antigenic and genetic heterogeneity of *T. maritimum* hindering development of effective biological products (e.g., vaccines), the available investigative techniques for isolates of interest within the *T. maritimum* population, including mPCR-serotyping and MLSA, represent a significant and robust advancement towards commercial vaccines.

## 4 Natural reservoirs and prevalence factors

The natural reservoir(s) of *T. maritimum* are unclear as little ecological data for this microorganism exist. However, the propensity of *T. maritimum* to survive for a prolonged time in different seawater microcosms (Avendaño-Herrera et al., 2006b), as well as its ability to infect different fish species indicates that water is an important infection route. This may explain why *T*. *maritimum* has been isolated from sediments, tank surfaces, and water exposed to infected fish stocks (Santos et al., 1999). Some reports suggest that natural *T. maritimum* outbreaks occur shortly after transferring fish from hatchery tanks to inshore net cages, could be explained by handling stress and/or skin damage, indicating pathogen-to-host access by horizontal transmission in seawater (see Avendaño-Herrera et al., 2006a).

Certain bacteria may use the ecosystem along with the normal microbiota of marine organisms as reservoirs (Higgins, 2000), as has been described for *T. maritimum*. To this end, jellyfish (*Phialella quadrata*) blooms could conceivably provide both a colonization site (via initial nematocyst-related injuries) and a source of *Tenacibaculum* infection (Ferguson et al., 2010). Barker et al. (2009) also suggested that the sea lice (*Lepeophtheirus salmonis*) might serve as an organic substrate capable of extending *Tenacibaculum* persistence in seawater. More recently, Llewellyn et al. (2017) tracked the diversity and composition of *S. salar* skin surface microbiota throughout a complete *L. salmonis* infection cycle, finding that the *Tenacibaculum* genus was perhaps the most abundant taxon across all mucus and water samples. Indeed, the ability of *T. maritimum* like member of *Flavobacteriaceae* family to survive in host skin mucus and cope with the bactericidal activity of the skin mucus reflects the potential of this tissue to act as a major reservoir (Magariños et al., 1995; Staroscik and Nelson, 2008; Mabrok et al., 2016; Guardiola et al., 2019).

One factor that plays a key role in the presence of *T. maritimum* is seawater temperature. Acute and/or chronic exposure to suboptimal temperatures is generally suppressive, especially for the adaptive immunity of fish, thus increasing disease prevalence (Abram et al., 2017). A significant rise in the severity and prevalence of tenacibaculosis has been reported at higher seawater temperatures and salinities (see Avendaño-Herrera et al., 2006a). Other evidence reported by Yamamoto et al. (2010) demonstrates the influence of water temperature on the experimental induction of tenacibaculosis in Japanese olive flounder (*Paralichthys olivaceus*) using immersion and dilution methods. These authors observed high mortality rates between 17 and 26 °C but not below 17 °C or above 26 °C. In contrast, Brosnahan et al. (2019) declared that warm seawater temperatures have no influence or strong correlation with an increased prevalence of *T. maritimum*. In turn, the rising seawater temperature around Tasmania has increased tenacibaculosis frequency (van Gelderen et al., 2009a), but, interestingly, massive tenacibaculosis outbreaks have also been recorded during the winter (Soltani et al., 1996; Olsen et al., 2011). Conversely, Abd El-Galil and Hashem (2012a) reported high disease prevalence in black damsel (*Neoglyphidodon melas*), Picasso triggerfish (*Rhinecanthus assasi*), and broomtail wrasse (*Cheilinus lunulatus*) during winter but no *T. maritimum* infections during summer. Likewise, López et al. (2010) reported three outbreaks of tenacibaculosis in wedge sole fish (*Dicologlossa cuneata*) cultured at 20.5°C in southwestern Spain. The winter water temperature ranged mainly from 15-20°C, which, as concluded by the authors, is perfect for the growth and propagation of *T. maritimum*. Taken together, these reports could explain the explosive appearance of tenacibaculosis outbreaks worldwide, since, as is known, climate change has increased the temperature of seawater globally (Marcogliese, 2008). Further studies are required to elucidate this issue further and assess the risks associated with this and other environmental parameters.

In addition to water temperature, tenacibaculosis is often associated with poor management conditions (e.g., high rearing density, reduced or excessive feed administration, and/or mechanical damage of the skin and mucus barrier) (van Gelderen et al., 2011; Escribano et al., 2020). Adverse growing conditions increase the intensity and distribution pattern of the causative agent in various organs of hosts (Cepeda and Santos, 2002). On the other hand, natural outbreaks of tenacibaculosis at farms without a prior history of *T. maritimum* infection imply that transferred fish may act as asymptomatic vectors. This is consistent with the proposed horizontal transmission of tenacibaculosis, as confirmed by van Gelderen et al. (2010a). Transmission of *T. maritimum* through seawater and directly from one host to another have been proposed as routes of infection (Fringuelli et al., 2012). Likewise, the successful recovery of bacteria from the intestine of apparently healthy infected fish without any histological and pathological lesions reflects another potential reservoir for *T. maritimum* (Faílde et al., 2013). Given all these factors, *T. maritimum* has the potential to harm a wide range of wild, captive, and farmed fish species, making it a pathogen worthy of serious consideration as a threat to the global fish-aquaculture industry (Elanco, 2018).

## 5 Geographical distribution and host susceptibility

The geographical and host distribution of *T. maritimum* isolates is presented in depth by Avendaño-Herrera et al. (2006a). Importantly, and as mentioned in section 4 *Natural Reservoirs and Prevalence Factors*, *T. maritimum* is a mesophilic microorganism that grows well between 15 and 34°C, a range representative of aquatic environments in which fish are farmed (Suzuki, 2015). Temperature range can likewise vary when considering *T. maritimum* isolates from warmer-water fish species (Frisch et al., 2018a), even *T. maritimum* isolates from salmon and other cold-water species appear to have lower optimal temperatures. Although the optimum growth temperature under laboratory culture conditions is 30°C. Incubation temperatures from 15 to 18°C are probably suitable for most salmonid-related isolates. Despite the slow growth of cultures seeded directly from external lesions, this bacterium forms distinctive colonies with mutable rhizoid edges and adherence capacity on low-nutrient media containing sea salts (e.g., *Flexibacter maritimus* medium [FMM] and Marine Agar 2216) (Pazos et al., 1996). Marine agar supplemented with 50 µg/mL kanamycin has also been devised for the recovery and isolation of *T. maritimum* directly from skin ulcers (Frisch et al., 2018a). Underscoring the need for suitable culturing media, microbiology companies have developed an FMM broth^1^ as the medium of choice for laboratories without immediate access to a natural source of seawater.

These advancements in isolation and culturing mean that *T. maritimum* has now been retrieved and recognized in more geographies and hosts than those described up to 2006. In Europe, *T. maritimum* has been isolated in Italy from farmed tub gurnard (*Chelidonichthys lucerna* L.) and wild turbot (*Scophthalmus maximus*) (Magi et al., 2007); and in Norway from the lumpsucker cleaner fish (*Cyclopterus lumpus*) (Småge et al., 2016b). In Asia, the pathogen was retrieved from Korean olive flounder (*P. olivaceus*) (Jang et al., 2009). In Africa, this bacterium has been reported in Egypt in association with gilthead sea bream (*Sparus aurata*) and European sea bass (*D. labrax*) (Moustafa et al., 2014; Moustafa et al., 2015). *Tenacibaculum maritimum* has even extended beyond food-oriented fish to some valuable ornamental fish, including the Picasso triggerfish, black damselfish, and broomtail wrasse in Egypt (Abd El-Galil and Hashiem, 2011; Abd El-Galil and Hashem, 2012a; Haridy et al., 2015). In French Polynesia, this bacterium has been described as the cause of massive mortalities (up to 90%) among the tropical orbicular batfish (*Platax orbicularis*) (Lopez et al., 2022b). Despite significant efforts to remain free-of *T. maritimum* infections in Chile, which is the second largest producer of salmon after Norway, outbreaks of *T. maritimum* have been reported in farmed Atlantic salmon (Apablaza et al., 2017) and rainbow trout (Valdes et al., 2021), and outbreaks have likewise been reported in Canada among Sockeye salmon (*Oncorhynchus nerka*) (Nekouei et al., 2018). Susceptible hosts for *T. maritimum* and the respective geographic origins are briefly summarized in **Table 2**.

**Table 2:** Susceptible host species for *Tenacibaculum maritimum* and the respective geographic origin (updated since 2006).

Geographic origin	Susceptible host	Country	References
**Asia**	Olive flounder *Paralichthys olivaceus*	Korea	Jang et al. (2009)
**Europe**	Tub gurnard *Chelidonichthys lucerna*	Italy	Magi et al. (2007)
	Wedge sole *Dicologlossa cuneata*	South-western Spain	López et al. (2009)
	Lumpfish *Cyclopterus lumpus*	Norway	Småge et al. (2016b)
	Sand tiger shark *Carcharias taurus*	Italy	Florio et al. (2016)
	Atlantic salmon *Salmo salar*	Spain, Scotland, Ireland	Downes et al. (2018)
	European sea bass *Dicentrarchus labrax*	Turkey	Yardimci and Timur (2016)
**America**	Atlantic salmon *Salmo salar*	Chile	Apablaza et al. (2017)
	Sockeye salmon *Oncorhynchus nerka*	Canada	Nekouei et al. (2018)
	Rainbow trout *Oncorhynchus mykiss*	Chile	Valdes et al. (2021)
**Africa**	Black damselfish *Neoglyphidodon meles* Picasso triggerfish *Rhinecanthus assasi*	Egypt	Abd El-Galil and Hashiem (2011)
	Broomtail wrasse *Cheilinus lunulatus*	Egypt	Abd El-Galil and Hashem (2012a)
	Gilthead sea bream *Sparus aurata*	Egypt	Moustafa et al., 2015)
	European sea bass *Dicentrarchus labrax*	Egypt	Moustafa et al. (2014)
**Oceania**	Orbicular batfish *Platax orbicularis*	French Polynesia	Lopez et al. (2022b)
	Chinook salmon *Oncorhynchus tshawytscha*	New Zealand	Brosnahan et al. (2019)

## 6 Presumptive and confirmative diagnostic procedures for *Tenacibaculum maritimum*

The initial diagnosis of tenacibaculosis caused by *T. maritimum* was, for a long period, based on clinical signs associated with diseased fish. However, field experience indicates that assigning a specific diagnosis based solely on this criterion is quite difficult as the same signs could also be attributable to other pathogens (Fernández-Álvarez and Santos, 2018). Insufficiency as a diagnostic method becomes clearer knowing that different *Tenacibaculum* species can be found in the same lesion. Indeed, clinical signs from coinfections can be difficult to distinguish since information is lacking as to which pathogen is responsible for which sign of infection. For example, tenacibaculosis is commonly associated with external clinical signs in Atlantic salmon, but the lesions are very different when caused by *T. maritimum* (i.e., body and caudal fin lesions) or *T. dicentrarchi* (i.e., head and snout lesions) (Nowlan et al., 2020). An erroneous attribution for the cause of mortality is most worrying when considering that such diagnoses are used to substantiate metaphylactic antibacterial treatments to cages containing healthy, moribund, infected, and/or disease-carrying fish. Another common, yet controversial, practice within Chilean salmon farms is the diagnosis of death caused by *Tenacibaculum* spp. when signs of mixed infection with the intracellular, facultative fish pathogen *Piscirickettsia salmonis* are present. Prior to 2018, such cases were classified exclusively as piscirickettsiosis (Irgang and Avendaño-Herrera, 2022). These cases are now classified as infections caused by a “*Tenacibaculum*-complex.” For instance, the first isolation of *T. maritimum* together with *T. dicentrarchi* from Chilean-farmed Atlantic salmon occurred during a harmful algae bloom caused by *Pseudochattonella* spp. (Apablaza et al., 2017). Similar tenacibaculosis coinfections have been reported in salmon farms in Norway (Olsen et al., 2011; Småge et al., 2016b), Australia (Wilson et al., 2019), and Canada (Nowlan et al., 2021).

Several studies further indicate differences in the susceptibility of certain fish species to *T. maritimum* infection based on age, and lesions vary greatly between affected fish species (Chen et al., 1995). Crucial to remember is that within a few days, damaged tissues can significantly deteriorate from early- to advanced-stage ulceration (Bernardet et al., 1994). Weight also impacts susceptibility; fish weighing 2-80 g are more susceptible to acute infection, whereas fish over 100 g seem less susceptible (Avendaño-Herrera et al., 2006a). Further influencing infection severity and mortality rates are temperature, water parameters, rearing conditions, stress, and interfering parasitic infestations (Nowlan et al., 2021). In a presumptive diagnosis, the main macroscopic lesions associated with *T. maritimum* infection are external and include ulcerative skin, tissue necrosis, mouth erosion, tail and fin rots, and necrosis of the gills and eyes [see review by Bernardet (1998); Avendaño-Herrera et al., (2006a)]. All of these tissues are usually infected by *T. maritimum*, resulting in bacterial networks around dead surfaces, producing potent toxins and attenuating host defense mechanisms (van Gelderen et al., 2009b; Guardiola et al., 2019). The most common pathologic finding internally is paleness of the organs (Toranzo et al., 2005). However, a more serious *T. maritimum* infection in a gray mullet (*Mugil capito*) specimen showed moderate erythematous hemorrhaging at the base of the anal and pelvic fins and septicemic lesions manifested as severe congestion in the gills, kidney, and other visceral organs, with bloody hemorrhagic exudates (Abdel-Latif, 2013).

**Figure 1:**
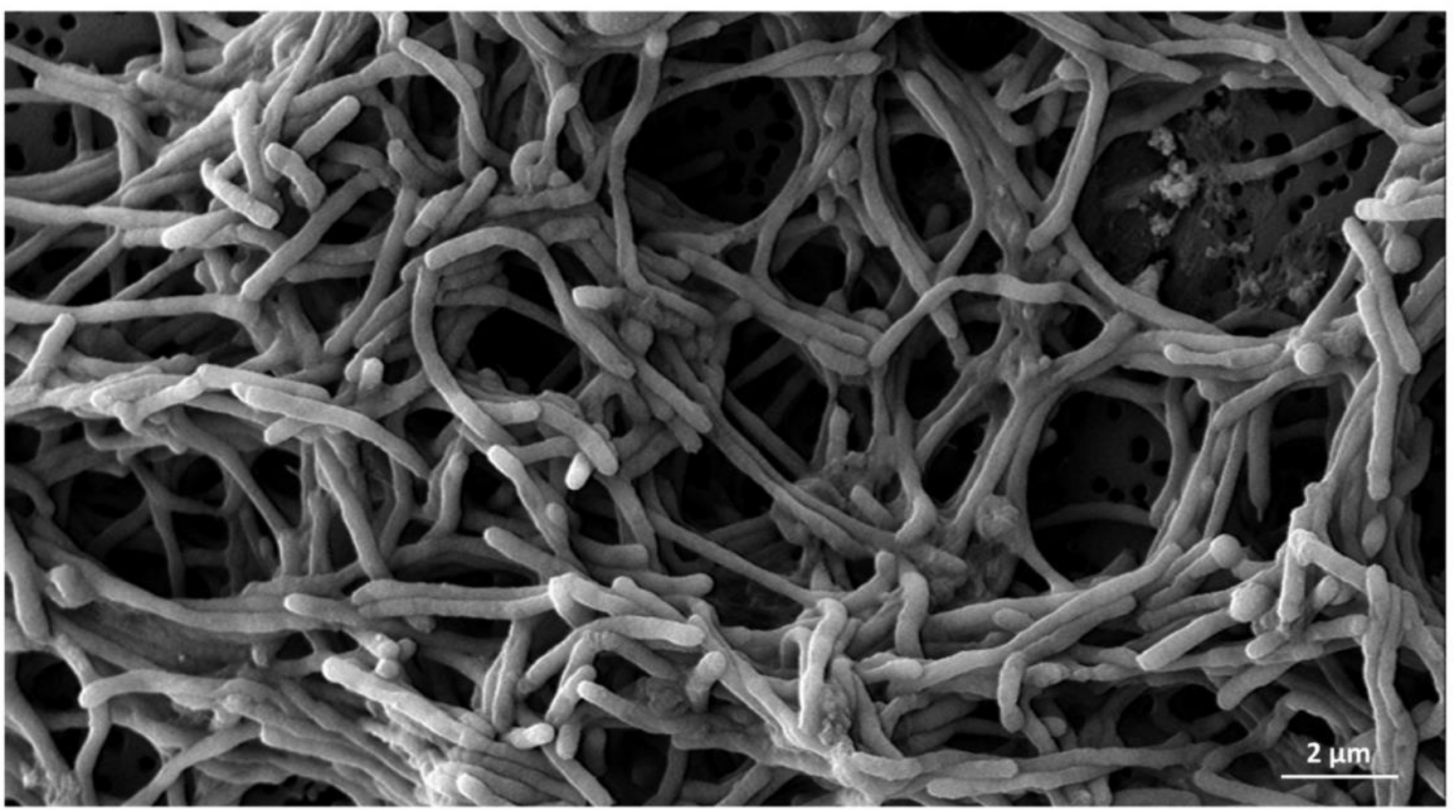
Scanning micrograph of the *Tenacibaculum maritimum* type strain CECT 4276^T^ at 96 hours of growth. The pathogen clusters are covered by a thick layer of biofilm and exopolysaccharides observed by scanning electron microscopy. The bar corresponds to 2 µm.

At the microscopic level, Vilar et al. (2012) described the pathologic changes in *S. sole* infected with *T. maritimum* using light and scanning-electron microscopy. The main lesions observed were detachment of the skin dermis and epidermis, hyaline degeneration, and necrosis in the musculature, as well as an inflammatory response around muscle cells. Moreover, scanning electron microscopy revealed the presence of filamentous, Gram-negative bacteria in the dermis layer and over the scales. Other research in a *P. orbiculares* specimen associated the intensity of ulcerative skin lesions with the number and area of whitish patches caused by *T. maritimum* (Lopez et al., 2022b). The respective histopathological view thereof for affected skin showed severe degeneration in both the dermis and epidermis layers with an aggregation of filamentous bacteria and leukocyte infiltration. These observations could be associated with the moderate hydrophobicity and rapid ability to form biofilms of *T. maritimum* isolates, as evidenced under *in vitro* conditions with live and dead cells in multi-layered bacterial aggregates (**Figure 1**). These traits may contribute to *T. maritimum* pathogenicity (Levipan et al., 2019). The aforementioned points complicate diagnosis and mean that macroscopic and microscopic observations are only a hypothetical approach. The use of laboratory protocols is, therefore, essential.

Definitive diagnosis requires colony isolation on specific media (i.e., FMM agar or media supplemented with antibiotics), but *T. maritimum* isolation from diseased fish is not always successful. Thereafter, minimal morphological and biochemical traits must be determined. *Tenacibaculum maritimum* typically display as flat, pale-yellow colonies with irregular edges on FMM agar, but on marine agar, colonies look round and yellow (Pazos et al., 1996). However, these traditional microbiological isolation and identification methods require a protracted time for precise diagnosis, resulting in high fish mortalities. These phenotyping assessments are becoming less frequently used due to the ever-increasing availability of advanced analytical methods (e.g., PCR, gene sequencing, and proteomic approaches). Moreover, specific molecular detection may be challenging given the diversity of undescribed *Tenacibaculum* taxa.

**Figure 2:**
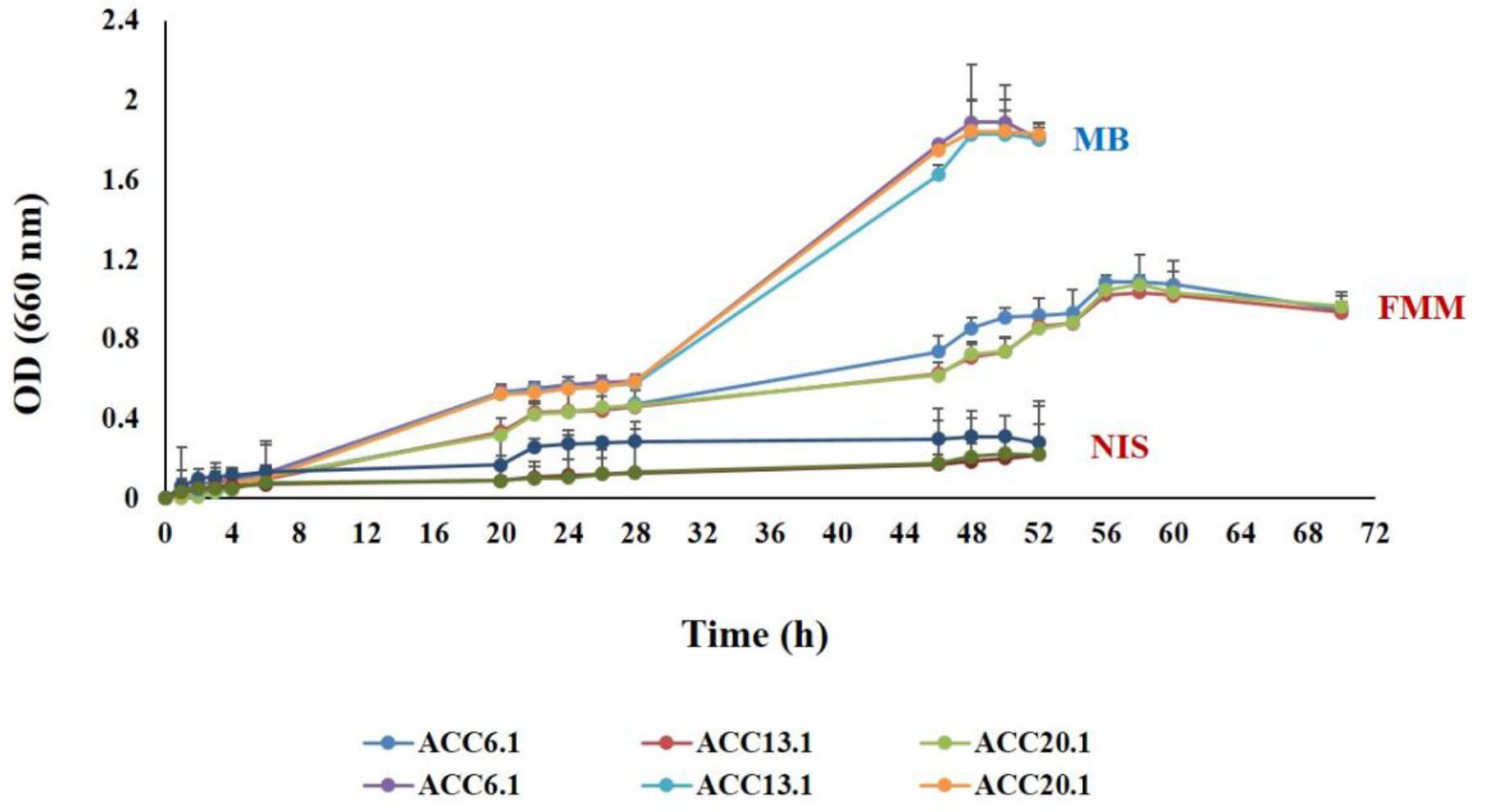
Comparative study to evaluate the bacterial growth curve of *T. maritimum* strains ACC20.1, ACC13.1, and ACC6.1 recovered from a Portuguese *Senegalese sole* farm following cultivation on marine broth (MB), marine broth treated with a ten-fold dilution of a detergent Igepal CA-630 (NIS), and *Flexibacter maritimus* broth (FMM). Data obtained from Abdelaziz (2016). Optical density (OD) readings were taken every 2 hours of incubation at 28°C and were performed in duplicate for each strain at 600 nm.

Importantly, *T. maritimum* as a filamentous bacterium presents many obstacles to laboratory work. Cells aggregate and adhere tightly to different substrates, thus impeding the recognition of *T. maritimum* colonies (Yamamoto et al., 2010). Mabrok et al. (2016) has evaluated several approaches to improve bacterial culture conditions, including treatment with non-ionic surfactants, detergents, cellulase hydrolysis, and shaking. Higher yields without clustering were obtained through continuous shaking during culturing (i.e., 140 rpm). This finding contrasts to other trials that gave obvious bacterial aggregates. However, bacterial growth depends on the employed culture medium. For example, significantly more limited growth is observed for FMM broth as compared to marine broth (**Figure 2**).

Undoubtedly, PCR protocols can serve as powerful tools to identify fish pathogens from plate cultures and fish tissues (Cunningham, 2002). The high sensitivity of the PCR technology promotes the early detection of pathogens soon after infection and even before disease onset. Since Toyama et al. (1996), various primer sets and PCR protocols (e.g., nested PCR or qPCR) have evolved for the specific detection of *T. maritimum* from pure or mixed cultures and fish tissues. Many protocols are based on the amplification of ribosomal genes, mainly 16S rRNA. All protocols are summarized in **Table 3**.

**Table 3:** PCR-based protocols described for species-specific detection of *Tenacibaculum maritimum*.

Gene of interest	Primers acronym	Designed primers 5′–3′	Processed sample	References
**Conventional PCR (C-PCR)**				
16SrRNA	F: MAR1 R: MAR2	AATGGCATCGTTTTAAA CGCTCTCTGTTGCCAGA	Pure and mixed cultures	(Toyama et al., 1996)
	F: Mar1 R: Mar2	TGTAGCTTGCTACAGATGA AAATACCTACTCGTAGGTACG	Pure and mixed cultures	(Bader and Shotts, 1998)
**Nested PCR**				
16SrRNA	F: 20F R: 1500R	AGAGTTTGATCATGGCTCAG GGTTACCTTGTTACGACTT	Pure and mixed cultures	(Cepeda and Santos, 2002)
	F: Mar1 R: Mar2	TGTAGCTTGCTACAGATGA AAATACCTACTCGTAGGTACG	Tissues infected fish	(Cepeda et al., 2003)
	F: pA R: pH	AGAGTTTGATCCTGGCTCAG AAGGAGGTGATCCAGCCGCA	Pure and mixed cultures	(Avendaño-Herrera et al., 2004c)
	F: MAR1 R: MAR2	AATGGCATCGTTTTAAA CGCTCTCTGTTGCCAGA	Tissues of challenged fish	(Avendaño-Herrera et al., 2004d)
**Quantitative real-time PCR (Q-PCR)**				
16SrRNA	F: MAR3 R: MAR6 Taqman probe	GATGAACGCTAGCGGCAGGC CCGTAGGAGTCTGGTCCGTG CACTTTGGAATGGCATCG	Pure and mixed cultures Tissues infected fish Formalin-fixed	(Fringuelli et al., 2012)
	F: Tm-rRNA16Fw R: Tm-rRNA16SFv	CTTTGGAATGGCATCGTTTT CGTAGGAGTCTGGTCCGTGT	Pure and mixed cultures	(Fernández-Álvarez et al., 2019)
β-actin (internal control)	F: β-actin-Fw R: β-actin-Rv	CTGAAGTACCCCATTGAGCAT CATCTTCTCCCTGTTGG CTTT	Tissues of naturally and experimentally infected fish	(Fernández-Álvarez et al., 2019)
Outer membrane protein A (ompA) gene	F: R: Probe:	GCCAATAGCAACGGGATACC TCGTGCGACCATCTTTGGT TGAATCAAATGCGATCTT	Tissues of experimentally infected fish	(Frisch et al., 2018b)

Other molecular methods that couple PCR amplification with a serological procedure or species-specific oligonucleotide probes are also available to detect *T. maritimum*, including the PCR-enzyme-linked immunosorbent assay (PCR-ELISA) (Wilson et al., 2002), reverse transcriptase polymerase chain reaction-enzyme hybridization assay (RT-PCR-EHA) (Wilson and Carson, 2003), and DNA microarray probe (Warsen et al., 2004). The incorporation of molecular probes allows pathogen detection in less time and with increased diagnostic specificity and robustness. Following assessments of unpublished *T. maritimum* genomes, Habib et al. (2014) notably proposed a genotyping scheme based on the multi-locus sequence analysis (MLSA). The resulting phylogenetic tree clearly distinguished *T. maritimum* from other relevant species, including *T. soleae*, *T. gallaicum*, *T. discolor*, *T. dicentrarchi*, and *T. ovolyticum*. Further revealed were the convoluted lineages between pathogenic and environmental strains, supporting the proposal of pathogenicity independently evolving in *Tenacibaculum* species (Habib et al., 2014; Bridel et al., 2018). Småge et al. (2016a) also propose other specific primers for the *atpD*, *fusA*, *pgk*, *rpoB*, and *tuf* genes, which could be used with isolates of other *Tenacibaculum* species (**Table 4**).

**Table 4:** Primer sets used for PCR analysis of *Tenacibaculum* species loci proposed in the MLST.

Locus	Primer Sequences (5′–3′)		Amplicon length (bp)	Reference
	Forward	Reverse		
***atpA***	ATTGGWGAYCGTCAAACWGG	CCAAAYTTAGCRAAHGCTTC	567	Habib et al. (2014)
***atpD***	TGGYCCAGTWATCGATGTTGA	AATACGYTCTTGCATTGCTC	807	Småge et al. (2016a)
***dnaK***	GGWACYACNAAYTCDTGTGT	TCWATCTTMGCTTTYTCAGC	573	Habib et al. (2014)
***fusA***	ATGGTAACTCACCCATTCCAGA	TGGCATGATGCAACACAAGG	767	Småge et al. (2016a)
***glyA***	CAYTTAACWCAYGGWTCDCC	ACCATRTTTTTRTTTACHGT	558	Habib et al. (2014)
***gyrB***	AGTATYCARGCRCTRGAAGG	GTWCCTCCTTCRTGYGTRTT	597	Habib et al. (2014)
***ileS***	CCWACHTTTGGWGCHGAYGA	GAATCRAACCAWACATCAAT	542	Habib et al. (2014)
***infB***	ATGCCDCAAACWAAAGARGC	GTAATHGCTCCAACYCCTTT	564	Habib et al. (2014)
***pgk***	GCTCCWCCACCWGTAGAAAC	GCTCCWCCACCWGTAGAAAC	935	Småge et al. (2016a)
***rlmN***	GCKTGTGTDTCDAGYCARGT	CCRCADGCDGCATCWATRTC	549	Habib et al. (2014)
***rpoB***	ATYTCTCCAAAACGCTGACC	AAAACGAATCAAGGWACGAAYA	3266	Småge et al. (2016a)
	ACCCTTTCCAAGGCATAAAGG	GAGCCATYGGTTTTGAAAGAGA		
	GAGCCATYGGTTTTGAAAGAGA	GAGCCATYGGTTTTGAAAGAGA		
***tgt***	GAAACWCCWATWTTYATGCC	TAYAWYTCTTCNGCWGGTTC	486	Habib et al. (2014)
***trpB***	GTWGCNCGWATGAAAATGYT	CCWGGRTARTCYAATCCTGC	368	Habib et al. (2014)
***tuf***	AGAGAWTTATTRTCTTTCTA	GTTACCTGACCWGCWCCWAC	554	Habib et al. (2014)
	ACCTCCTTCACGGATAGC	TTACGATCGTTCGAAGCCCC	971	Småge et al. (2016a)
***yqfO***	GCBGAARRTTTTGAYAAYGT	AYTTCRTARGCDACYTCTTC	446	Habib et al. (2014)

Multi-locus sequence analysis is now widely used not only for genetic differentiation and the estimation of evolutionary relationships among *Tenacibaculum* species, but also for the specific identification of *T. maritimum* from pure cultures (Småge et al., 2016a; Valdes et al., 2021; Lopez et al., 2022a). MLSA provides unambiguous DNA sequence data that can be readily compared through web-based databases globally, thus providing unbiased information similar to or better than that provided by other PCR-based methods (Lin et al., 2014). The recommended primer sets for MLSA are summarized in **Table 4**.

Most PCR protocols are (a) time-consuming; (b) liable to produce multi-sized and nonspecific amplicons for environmental and field samples; and (c) require thermal cycling devices (Klein, 2002; Yang and Rothman, 2004; Mabrok et al., 2021). A recent breakthrough – Matrix-Assisted Laser Desorption/Ionization-Time of Flight (MALDI-TOF) mass spectrometry (MS) – has risen as an alternative diagnostic tool for the in-field and laboratory detection of marine *Tenacibaculum* species (Fernández-Álvarez et al., 2017). A recent molecular study by Bridel et al. (2020) investigated the genomes of 25 *T. maritimum* isolates, the average genome sizes of which were ~3.356 Mb. The core genome involved 2,116 protein-coding genes (~75%). An adequate level of nucleotide diversity was observed in the most conserved domains (~0.0071 bp−1). The conducted molecular analyses further evidenced remarkable recombination (r/m ≥ 7) and categorization into various subgroups. Moreover, MALDI-TOF-MS was used to detect biodiversity among the recovered 25 *T. maritimum* strains. The sequence analyses revealed various mass peaks that resembled ribosomal proteins. Furthermore, variation in one or more amino acids was demonstrated, illustrating the mass shift in the bacterial ribosomal proteins. These detected peak shifts greatly associated with the specific genotype defined by MLSA. Consequently, both MALDI-TOF-MS and MLSA are proposed as techniques highly specific for the diagnosis of *T. maritimum* when pure bacterial isolates are studied.

## 7 Pathogenicity of *Tenacibaculum maritimum* infection

Pathogenicity is defined as the hereditary capacity of a microorganism to actuate infection, as interceded by particular harmful variables. Pathogenicity also alludes to the degree of bacterial destructiveness and harmful impact on host tissues (Casadevall and Pirofski, 2001). This concept is often associated with host susceptibility as well as the virulence of the causative agent (Brock et al., 2003). As presented in **Table 2**, the distribution of *T. maritimum* among fish species other than those indicated by Avendaño-Herrera et al. (2006a) has been confirmed. Indeed, *T. maritimum* shows a lack of strict host specificity, thus expanding previously described geographical and host distributions, as described in depth in section 5 *Geographical Distribution and Host Susceptibility*. Tenacibaculosis may therefore be a risk for many species of anadromous and marine fish, including in species in which the disease has not yet been described or, unfortunately, simply ignored. Controversy arises in cases where serotypes (Avendaño-Herrera et al., 2004a; Avendaño-Herrera et al., 2005a; Lopez et al., 2022a) and/or genotypes particular of *T. maritimum* strains (Habib et al., 2014) colonize certain species or are distributed in specific geographical areas.

For example, Avendaño-Herrera et al. (2006c) reported a sole isolate that does not produce mortality in turbot fry specimens challenged by intraperitoneal and immersion routes. Likewise, Frisch et al. (2018c) reported differences in the cumulative mortality of different Canadian isolates of *T. maritimum* against Atlantic salmon challenged by bathing. The inefficiency in reproducing *T. maritimum* infection under laboratory conditions using a single infection model (i.e., injection, cohabitation, or immersion) supports scientific research that *T. maritimum* could be an opportunistic pathogen that primarily causes extensive skin damage and gill abrasion with subsequent systemic infection. Current scientific evidence supports disease transmission through seawater, but limitations presented by the initial variability of inoculum of *T. maritimum* during growth in microbiological cultures – that is, the formation of bacterial aggregates (Mabrok et al., 2016) – could affect success in the natural simulation of *T. maritimum* infection.

Initial pathogenicity studies conducted in the 1990s with commercial fish species such as European sea bass (Bernardet et al., 1994), rainbow trout, greenback flounder (*Rhombosolea tapiriña*) (Soltani et al., 1996), and Atlantic salmon (Powell et al., 2004) found varying degrees of mortality based on infection method and routes of bacterial inoculation, as noted above. By contrast, Avendaño-Herrera et al. (2006c) reported that the tested *T. maritimum* isolates (regardless of dosage or serotype) did not cause disease when injected intraperitoneally but did cause significant mortality in turbot after an18 h bath challenge. This first approach to establishing a robust challenge model using advances in knowledge of *T. maritimum* virulence properties has resulted in decreasing bath-challenge exposure times to just a few hours (i.e., 1.5 to 7 h of incubation) (Frisch et al., 2018c; Valdes et al., 2021).

The available scientific evidence shows that seawater is of crucial importance in the horizontal transmission of *T. maritimum*, but other routes of entry to the host cannot be ruled out, such as direct contact with marine invertebrates colonized by the bacterium (Ferguson et al., 2010). This includes through food, as reported by Chen et al. (1995). Environmental factors (i.e., seawater temperature and salinity) further influence the severity of *T. maritimum* infections, but more exhaustive studies are required to precisely identify the primary reservoirs of infection and/or the role of culture water in the epidemiology of infections and specificity of risk factors. This compilation of knowledge is needed to control and prevent *T. maritimum* outbreaks.

## 8 Virulence factors

Virulence is the relative capacity of a microorganism to cope with the host’s immune response and to replicate and reproduce, thus resulting in disease development (Casadevall and Pirofski, 1999). In the context of this review, virulence is the degree to which *T. maritimum* can produce tenacibaculosis. A first step for the effective colonization of a pathogenic microorganism within a host is adherence. Interestingly, the name of the bacterial genus [i.e., Te.na.ci.ba’cu.lum. L. adj. n. *tenax -acis* holding fast; L. neut. n. *baculum* stick; N.L. neut. n. (Suzuki et al., 2001)] indicates that *Tenacibaculum* species are rod-shaped bacteria that adhere to the surface of marine organisms. There are two forms of adhesion, specific and non-specific. These adhesion forms allow microbial expansion within host tissues and encourage the dispersion of toxins (Ofek and Doyle, 2012). Specific adhesion is mediated by certain compounds on the bacterial surface that bind to receptors on host tissues, and non-specific adhesion depends on hydrophobic or ionic bonds between the bacterial surface and the supporting substrate (Ofek and Doyle, 1994).

Burchard et al. (1990) reported that the adhesion power of *T. maritimum* cells is increased constitutivelyon substrates with low-critical energy surfaces (e.g., hydrophobic). This was ascribed to the capacity of the microbes to deliver extracellular polymers or slime, which permit a solid connection to hydrophobic surfaces. Most invading pathogens recognize and bind to fish mucus through sugar-binding proteins such as lectins. This is the first step in initiating defensive responses (Imberty and Varrot, 2008) since the abundance of carbohydrate residues in the skin mucus of hosts has been related to the progression of infection (Estensoro et al., 2013). This hallmark reflects the capacity of pathogens to attach to the external surfaces of many fish species. This hypothesis was affirmed by Magariños et al. (1995), who stated that *T. maritimum* adheres strongly to the skin mucus of turbot, sea bream, and European sea bass, regardless of the salt solution in which the mucus dissolves.

Moreover, *in vitro* work established by van Gelderen et al. (2010b) proved the adhesive nature of different *T. maritimum* strains. Similarly, data regarding mucus and plasma bactericidal activities revealed a considerable lack of immunity in *S. sole* against *T. maritimum* and proved the ability of this pathogen to present evading strategies against the infected host (Mabrok et al., 2016). Even in turbot that survived exposure to virulent strains, *T. maritimum* was only recovered from mucus (Avendaño-Herrera et al., 2006c). The same team of researchers subsequently showed that *T. maritimum* is part of the autochthonous bacterial population in fish and can survive for long periods in the aquatic environment by using fish mucus as a reservoir (Avendaño-Herrera et al., 2004c; Avendaño-Herrera et al., 2006d). In the case of *T. maritimum*, this adhesion and colonization capacity cannot be attributed to bacterial structures such as pili, fimbriae, or flagella since *T. maritimum* cells lack all of these elements (Avendaño-Herrera et al., 2006a). Guardiola et al. (2019) found that *T. maritimum* can withstand and overcome the skin mucus barrier due to a passive change of the glycosylation pattern.

Besides stickiness, the hemagglutinating activity of *T. maritimum* may also influence its destructiveness. Further demonstrated by Pazos (1997), was that *T. maritimum* agglutinates different types of blood cells. Extracellular products, subcellular components, and lipopolysaccharides are additional virulence-related mechanisms of *T. maritimum*, as detailed in depth in the review by Avendaño-Herrera et al. (2006a). The bacterial toxins present in extracellular products may also play a role during disease either by encouraging bacterial colonization and invasion or by changing and disintegrating host tissues (Baxa et al., 1988). Supporting these points, van Gelderen et al. (2009b) demonstrated that *T. maritimum* toxins induce gill necrosis, cellular damage in the heart and pyloric caeca, and autolysis and rapid lysis in *S. salar* cells.

*T. maritimum* is a proteolytic fish pathogen (Wakabayashi et al., 1986). The release of extracellular proteases is an index of virulence, with effects exerted on the tissues and defense mechanisms of the host. In this sense, the production of toxins and proteolytic enzymes during bacterial invasion is essential for successful colonization and virulence (Rahman et al., 2014). According to Frisch et al. (2018b), *T. maritimum* proteases act synergistically with other virulence determinants, ultimately resulting in tissue damage or mortality for the host. The involvement of bacterial toxins or extracellular enzymes in the invasion strategy of and successful generalized infection by *T. maritimum* was further supported through observations of a delayed mucosal immune response and incipient plasma peroxidase and lysozyme activities observed in bath-challenged *S. senegalensis* (Guardiola et al., 2019).

**Figure 3:**
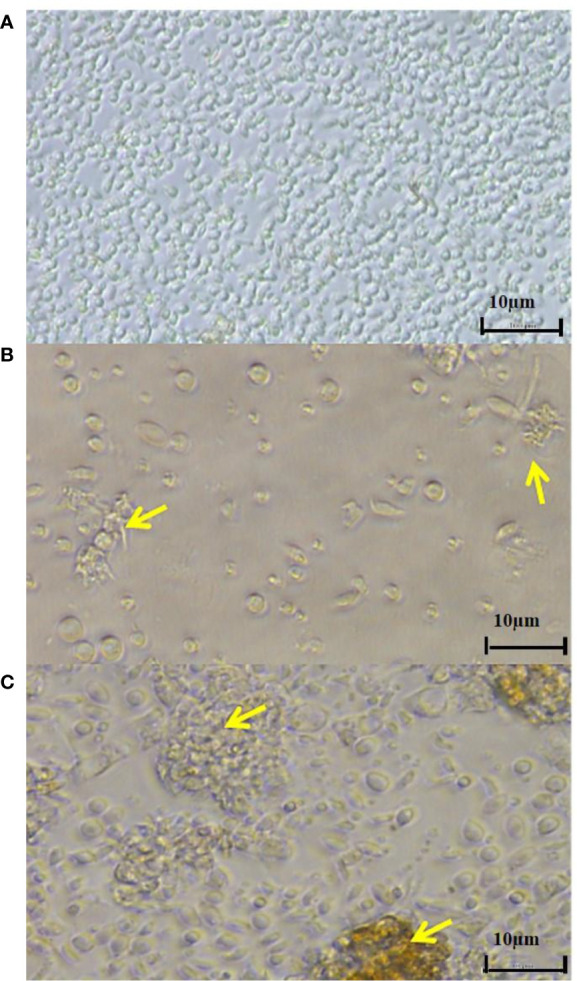
Microscopic pictures of *Senegalese sole* head-kidney leukocytes after exposure to **(A)** L15 medium or **(B, C)** extracellular products of *T. maritimum* at 100 µg mL^-1^ for 24 h. Yellow arrows indicate **(B)** inner vacuolization, cell elongation and/or degradation and **(C)** cell clustering and conglomeration. These results were obtained from Abdelaziz (2016).

Despite efforts made in the past few decades, the pathogenesis of *T. maritimum* is a multifactorial process that is still not fully understood. To further elucidate the cell-level immune responses of and the cytotoxic effects against *S. senegalensis* head-kidney leukocytes, Abdelaziz (2016) performed *in vitro* trials through exposure to the extracellular products of different *T. maritimum* isolates. The leukocytes increased the production of reactive oxygen species but not of nitric oxide. Interestingly, while a low concentration of *T. maritimum* extracellular products triggered production of reactive oxygen species, a high concentration suppressed production; thus reflecting the ability of *T. maritimum* to cope with the phagocytic response of *S. senegalensis* using suppressive toxins or enzymes (Abdelaziz, 2016). Cytotoxic activity against the leukocytes was likewise dose-dependent, providing initial clues into the mechanisms by which *Tenacibaculum* actuate cell death. To this end, light microscopy of fish leukocytes showed inner vacuolization, cellular detachment and prolongation, cell-membrane weakening, and cell clustering and conglomeration, especially after incubation with high concentrations of extracellular *T. maritimum* products (**Figure 3**) (Abdelaziz, 2016).

One of the most studied virulence mechanisms of *T. maritimum* is its ability to compete effectively with the host for iron (Ratledge and Dover, 2000). Avendaño-Herrera et al. (2005b) suggested at least two systems of iron acquisition for different *T. maritimum* serotypes: one involving the synthesis of siderophores and another allowing for the utilization of heme groups as iron sources by direct binding. These physiological results are supported by molecular studies that demonstrate a siderophore-biosynthesis gene cluster (*tbs* gene) in the *T. maritimum* genome. This gene cluster is responsible for the synthesis of the bisucaberin-class siderophore (Fujita et al., 2012). The *tbs* genes are encoded to produce *Tenacibaculum* bisucaberin synthase. Moreover, various TonB-dependent outer membrane receptors were detected in the *T. maritimum* genome; the *MARIT*_0185 type is placed in the tbs locus that encodes for the bisucaberin siderophore iron transporter. The genome of *T. maritimum* has genes encoding proteins that initiate iron uptake and/or storage (Pérez-Pascual et al., 2017). As previously described, *T. maritimum* is characterized by non-flagellar gliding motility that enables movement over various surfaces. It is no surprise then that the *T. maritimum* genome contains 14 gliding genes (*gldA* to *gldN*) and 10 *spr* genes that encode for various proteins needed for the gliding motility (Pérez-Pascual et al., 2017). Pérez-Pascual and colleagues (2017) additionally detected numerous genes for adhesins and polysaccharide biosynthesis. The proteins displaying lectin or carbohydrate-binding motifs could be involved in the strong adhesive and hemagglutination properties, as well as biofilm-forming ability, of *T. maritimum* described by Pazos (1997). Surface-attached biofilms and cell-surface hydrophobicity are fundamental for the environmental persistence and transmission of *T. maritimum* (Burchard et al., 1990). The kinetics of biofilm formation in *T. maritimum*, especially on adhesive surfaces may serve as transient reservoirs of virulent strains that can further exacerbate the outbreak of tenacibaculosis after the cell-detachment stage (Levipan et al., 2019).

The *T. maritimum* genome also has genes encoding the superoxide dismutases manganese-dependent type (*sodA*), iron-dependent *sodB*, and zinc-dependent type (*sodC*), the last of which is only found in virulent strains (Sheng et al., 2014). These enzymes enable the bacteria to transform superoxide anions into molecular oxygen and hydrogen peroxide that can be easily metabolized by peroxidase and catalase enzymes, the end effect of which is tolerance to the oxidative stress exerted by the host immune response (Lee et al., 2012). Similarly, these enzymes act as a group of powerful toxins and virulence determinants mainly involved in damaging host cells, including ceramidase and a sphingomyelinase (Pérez-Pascual et al., 2017). Sphingomyelinase has been described as a highly potent cytotoxin to the host (Oda et al., 2010), and ceramidase acts as a potent exotoxin (Ito et al., 2014). Numerous other genes associated with virulence mechanisms in *T. maritimum*, especially toxins, were summarized in depth by Pérez-Pascual et al. (2017). These genes include a cholesterol-dependent cytolysin, an alternative hemolysin gene, and the *cslA* gene (*MARIT_2107*) encoding a PL8_3 family chondroitin AC lyase. These were very similar to those produced by another fish pathogen, *Flavobacterium columnare* (Suomalainen et al., 2006), and each plays an invasive role in fish tissues.

Not all genetic information on *T. maritimum* has reached consensus. For example, controversy surrounds the existence of communication processes mediated by quorum sensing. Romero et al. (2010) identified n-butyryl-L-homoserine lactone (C4-HSL) by liquid chromatography-mass spectrometry in the type strain of *T. maritimum* and demonstrated degradation activity for long N-acyl-homoserine lactones (C10-HSL). By contrast, Pérez-Pascual et al. (2017) did not detect a homologous gene for homoserine lactone biosynthesis in the genome, nor were any coding genes found for processes involved in the inhibition of bacterial communication (i.e., quorum quenching).

The genome sequences of the *T. maritimum* type strain provided valuable insights into the existence of many predicted genes potentially implicated in the virulence and pathogenicity of *T. maritimum*. Through the analysis of 24 *T. maritimum*-strain genomes recovered from different hosts and geographical areas, Bridel et al. (2020) identified all the predicted toxins in the core-genome and the virulence factors (i.e., cholesterol-dependent cytolysin, collagenase, sphingomyelinase, ceramidase, chondroitin AC lyase, streptopain family protease, sialidase, iron uptake systems, and type IX secretion system [T9SS]) previously identified by Pérez-Pascual et al. (2017). Continued confirmations through physiological studies of the initial work by Pérez-Pascual and colleagues (2017) will help elucidate and describe the genes and pathways likely involved in *T. maritimum* virulence. Therefore, further physiological research is necessary to understand the pathogenesis of tenacibaculosis.

## 9 Treatment and vaccination programs for *Tenacibaculum maritimum*

To date, the only commercially available vaccine for *T. maritimum* is for turbot, specifically strain LPV1.7 serotype O2^2^. Hence, the control of tenacibaculosis in other cultured species and for other strains is mainly limited to the frequent use of antibiotics and certain disinfectants (Avendaño-Herrera et al., 2008). Antibiotics can be used to treat tenacibaculosis with varying degrees of success. As a preventive or prophylactic measure, surface-acting disinfectants administered by immersion may be effective. Temperature and/or salinity manipulation may also aid in disease mitigation (Santos et al., 2019). Notwithstanding, the susceptibility of tenacibaculosis-causing agents to different antimicrobials commonly used in aquaculture is highly variable and dependent on *Tenacibaculum* species and the antimicrobial.

An *in vitro* study using disk diffusion susceptibility testing on FMM plates, showed that 63 *T. maritimum* isolates from several hosts and geographical regions were resistant to oxolinic acid but susceptible to amoxicillin, nitrofurantoin, florfenicol, oxytetracycline, and trimethoprim-sulfamethoxazole (Avendaño-Herrera et al., 2008). In addition, some isolates presented resistance to enrofloxacin and flumequine. A previous study – the goal of which was to propose a standardized culture medium and procedure for determining the minimum inhibitory concentration – showed that 32 *T. maritimum* strains were resistant to oxolinic acid but highly susceptible to amoxicillin and trimethoprim-sulfamethoxazole (Avendaño-Herrera et al., 2005c). More recently, Abdelbaky et al. (2021) found that all *T. maritimum* strains examined from diseased Haffara bream (*Rhabdosargus haffara*) and marbled spinefoot (*Siganus rivulatus*) were sensitive to erythromycin, cephalothin, ampicillin, and chloramphenicol but showed high resistance to ofloxacin and tetracycline. More confounding is that field results are not always the same, even if the tested bacteria show a higher sensitivity to the chosen drug *in vitro* (Cepeda and Santos, 2002).

The widespread use of antimicrobials in aquaculture could cause the appearance of multidrug-resistant *T. maritimum* strains (Watts et al., 2017). In the related fish pathogen, *Flavobacterium psychrophilum*, R-plasmids, antimicrobial resistance genes, and mutations in resistance elements are associated with antibiotic resistance (Izumi and Aranishi, 2004). However, *T. maritimum* does not have plasmids. Therefore, the occurrence of antimicrobial resistance could be associated with some intrinsic resistance mechanism, such as the efflux pump system, permeability of the bacterial outer cell membrane, and/or the synthesis and release of antibiotic-degrading enzymes, especially β -lactamase (Clark et al., 2009; Henríquez-Núñez et al., 2012; Pérez-Pascual et al., 2017).

Enrofloxacin, a second generation fluoroquinolone, usually exhibits promising antimicrobial activity against *Flavobacterium* spp. (Akinbowale et al., 2006). Two new antimicrobial 2-alkyl-4-quinolones, a common core in synthetic antibactericidals, showed promising antibacterial activity against *T. maritimum*, thus providing a new opportunity to develop antibacterial drugs for fish farming (Li et al., 2018). However, the frequent use of these compounds for tenacibaculosis treatment has decreased efficacy and resulted in futile usage (Jang et al., 2009). Another widely applied antimicrobial in aquaculture worldwide is florfenicol, a compound specifically developed for veterinary medical use. Florfenicol is widely used for the treatment of tenacibaculosis caused by the different species of the genus (Irgang et al., 2021). Interestingly, the existence of a multidrug efflux pump present in *T. maritimum* has been reported (Pérez-Pascual et al., 2017), which could cause resistance to florfenicol. Such resistance has been previously reported in *Chryseobacterium* species, another member of the *Flavobacteriaceae* family (Michel et al., 2005). These efflux pumps also play a key role in intercellular signaling, bacterial virulence, and detoxification of intracellular bacterial metabolites (Pasqua et al., 2019). Another widely used antimicrobial in fish aquaculture, particularly in developed countries, is oxytetracycline (Avendaño-Herrera et al., 2023). Yet again, extensive usage may soon result in efficacy deterioration (Irgang et al., 2021). Considering the current scenario for global aquaculture, several actions are required to prevent the emergence of multidrug-resistant bacterial pathogens and maintain the efficacy of antibiotics – (i) the continuous monitoring and surveillance of the antimicrobial susceptibility of *T. maritimum* isolates using laboratory tests, such as those proposed by the Clinical Laboratory Standards Institute^3^ for bacteria isolated from aquatic animals and (ii) the proper use and rotation of antimicrobial agents.

The emergence of multiple drug resistant pathogens, together with the negative impact of antibiotics on the beneficial gastrointestinal microbiota of fish, has encouraged developed countries to prohibit the use of all subtherapeutic antibiotics (Regulation [EC] No 1831/2003, Europe) (Simon and Klaus, 2008). This has pushed the scientific community to explore non-antibiotic related alternatives, such as probiotics and treatment with herbal dietary medications. For example, Reyad et al. (2013) demonstrated that the marine actinomycetes probiotic (YSCl2334) – isolated from Salwa Beach in the Jazan Region of Saudi Arabia – can generate antimicrobial activity against *T. maritimum*. Likewise, the probiotic *Roseobacter* group (*Phaeobacter piscinae* S26) can be effective in killing pathogenic *Tenacibaculum* species, including *T. maritimum* (Tesdorpf et al., 2022). Similarly, twelve indigenous cultured probiotic isolates from the gastrointestinal tract of three temperate flatfish species (Wanka et al., 2018), as well as four spore-former isolates obtained from the heat-treated gut contents of European sea bass (Serra et al., 2019), showed promising inhibition activity towards **T. maritimum*.*

Comparably, plant extracts exhibit potent antimicrobial activity against a variety of Gram-positive and Gram-negative bacteria and are generally regarded as safe by the Food and Drug Administration (Soniya et al., 2013). To this end, *in vitro* susceptibility studies indicated potential antimicrobial activity of carvacrol, a derivative of the oregano plant (Abd El-Galil and Hashiem, 2012b), and of some native plant extracts from Jeju Island against tested *T. maritimum* strains (Jang et al., 2009). All these data provide insights into the potential use of non-antibiotic compounds in treating and preventing the pathogenic threat of *T. maritimum*; however, further research into the applications and appropriate dosages thereof is still being conducted.

Despite recent strategies applied for effective treatment, bacterial fish diseases remain a major economic obstacle to aquaculture globally. The emergence of multidrug-resistant bacterial pathogens increases the need for effective vaccination programs (Sneeringer et al., 2019). Modern vaccine technology targets specific bacterial component, such as novel antigens (Cimica and Galarza, 2017). RNA particle-vaccines provide greater immunity as compared to conventional protocols. Innovations in vaccine manufacturing are indeed promising for the aquaculture industry (Frietze et al., 2016). However, few attempts at commercial-level vaccinations have been described, and, as previously mentioned, only one bacterin is available on the market for turbot (Romalde et al., 2005). The developed vaccine was applied by bathing for fish of 1-2 g, followed by a booster injection dose at a size of 20-30 g. The survival rate of the challenged fish after bath immunization was about 50%, and increased to >85% after booster injection. The systematic application of this vaccine in some turbot farms has decreased the prevalence of tenacibaculosis.

Numerous vaccination trials in salmonids exist, but with contradictory outcomes. The first trials of a *T. maritimum* vaccine for Atlantic salmon farmed in Australia resulted in little or no protection (Carson et al., 1993; Carson et al., 1994). However, *S. salar* immunized with an adjuvant-based vaccine performed better and showed a greater survival rate than other tested vaccines at 27 days post-challenge with *T. maritimum* (van Gelderen et al., 2009a). More recently, an inactivated whole-cell oil-adjuvant vaccine against *T. maritimum* produced an antibody response in Atlantic salmon, but with no significant protection (Frisch et al., 2018c). The antigenic and genetic variability existing among *T. maritimum* isolates complicates candidate selection for vaccine development. Supporting this, Salati et al. (2005) emphasized that lipopolysaccharides are the main protective antigens of this pathogen.

To date, no commercial vaccine against tenacibaculosis in salmonids is available, and Toranzo et al. (2004) indicate that the vaccine developed for turbot is not effective in preventing tenacibaculosis in other fish species. Autovaccine or autogenous bacterins are alternatives that several scientists havedeveloped and implemented. However, important information gaps exist regarding the effectiveness of these biological products and the host defense mechanisms. These factors altogether may hinder the development of appropriate vaccination programs for this economically impactful *Tenacibaculum* species.

## 10 Conclusion and knowledge gaps

Tenacibaculosis has risen over the past five years to become one of the most devastating bacterial infections impacting various species of farmed fish and geographical regions. The abrupt re-emergence of disease caused by *T. maritimum* is in addition to the appearance of new *Tenacibaculum* species, currently placing the global salmonid aquaculture industry at risk. Considering the broader availability of scientific studies on *T. maritimum* in the last 15 years, we conducted an extensive review on the status of and advancements on this bacterium since 2006. In summary, the taxonomic status of this bacterium is clear, consensus exists regarding intra-specific diversity, and specific, sensitive diagnostic tools are available (i.e., PCR, molecular probes in PCR-ELISA, RT-PCR-EHA, and MALDI-TOF assays, etc.) that are applicable to pure cultures as well as in early detection through infectious signs occurring in farmed fish. Advancements in genome sequencing have paved the way for developing methods invaluable to the genetic (i.e., MLSA) and serotype (i.e., mPCR) tracking of *T. maritimum*. Both tools could be useful in creating new species-specific vaccines. Other significant advancements include those related to disease replication under laboratory conditions, specifically through the immersion of fish with the pathogen, thus simulating natural conditions. This tool is indispensable in the search for new products for the treatment (e.g., plant-based compounds) and prevention (e.g., vaccines) of tenacibaculosis. Unfortunately, understanding remains lacking regarding the reservoirs for this bacterium and for the risk factors that influence bacterial appearance. Particularly unclear is the influence of environmental factors (e.g., seawater temperature), an important point considering the context of globally increased temperatures due to climate change. The genome has provided information on the existing mechanisms of pathogenicity in *T. maritimum*, but physiological studies demonstrating the participation of proteins, and not just the presence of the gene, are deficient. Research has also advanced knowledge of the fish response to *T. maritimum*, but much remains to be explored. This situation is likely the driver behind antibiotics remaining the most used measure to control *T. maritimum*. As such, there is an urgent need to establish best-practice sanitary measures, to develop new vaccines and autovaccines, and to search for other control measures besides antibiotics, which potentially include herbal-based medications, phages, or probiotics. Lastly, more research is needed to determine why *T. maritimum* and other *Tenacibaculum* species are generally considered opportunistic pathogens. This point takes on particular relevance when considering that some *Tenacibaculum* isolates possess a larger virulence arsenal than other isolates, as specifically due to the diversity within this bacterial genus. Consequently, we must be open to understanding that fish are farmed intensively, and bacterial infections are not restricted and specific to a single microorganism. Rather, coinfections occur, whether with different bacteria or, in many cases, with the same bacterial species but of different genotypes/serotypes. The presence of *T. maritimum* or other *Tenacibaculum* species in the aquatic environment can therefore be a factor in the health of farmed fish, regardless of the pathogen being primary, secondary, or, simply, a coexisting microorganism. Future research should focus on resolving the pending questions raised by our review.

## Author contributions

MM, AA, HH, ES, AF and RA-H participated in writing and editing of the manuscript. BA, SA and RA-H constructed the contents of the figures and tables. CR and RA-H participated in and supervised the thorough review of the manuscript. All authors contributed to the article and approved the submitted version.

## Footnotes

1
https://www.condalab.com/medios-de-cultivo-deshidratados/1050-5471-caldo-fmm.html#/2-formato-500_g

2
https://www.hipra.com/wcm/connect/hipra/7c54199f-880a-43bc-a46e-4e981deaad2f/ICTHIOVAC-TM-ETI-701222-01.4.pdf?MOD=AJPERES&amp;CACHEID=ROOTWORKSPACE.Z18_L26A0J40OGJR60QGTTTS0N3067-7c54199f-880a-43bc-a46e-4e981deaad2f-mSMeSVA

3
www.clsi.org
